# Supplementary material for: Breathing coordinates cortico-hippocampal dynamics in mice during offline states
Source: Nat Commun. 2022 Jan 24;13:467. doi: 10.1038/s41467-022-28090-5 (PMC8786964; doi:10.1038/s41467-022-28090-5)
Supplement: Supplementary file 1 — Supplementary Information [file 41467_2022_28090_MOESM1_ESM.pdf]

Supplementary Figures

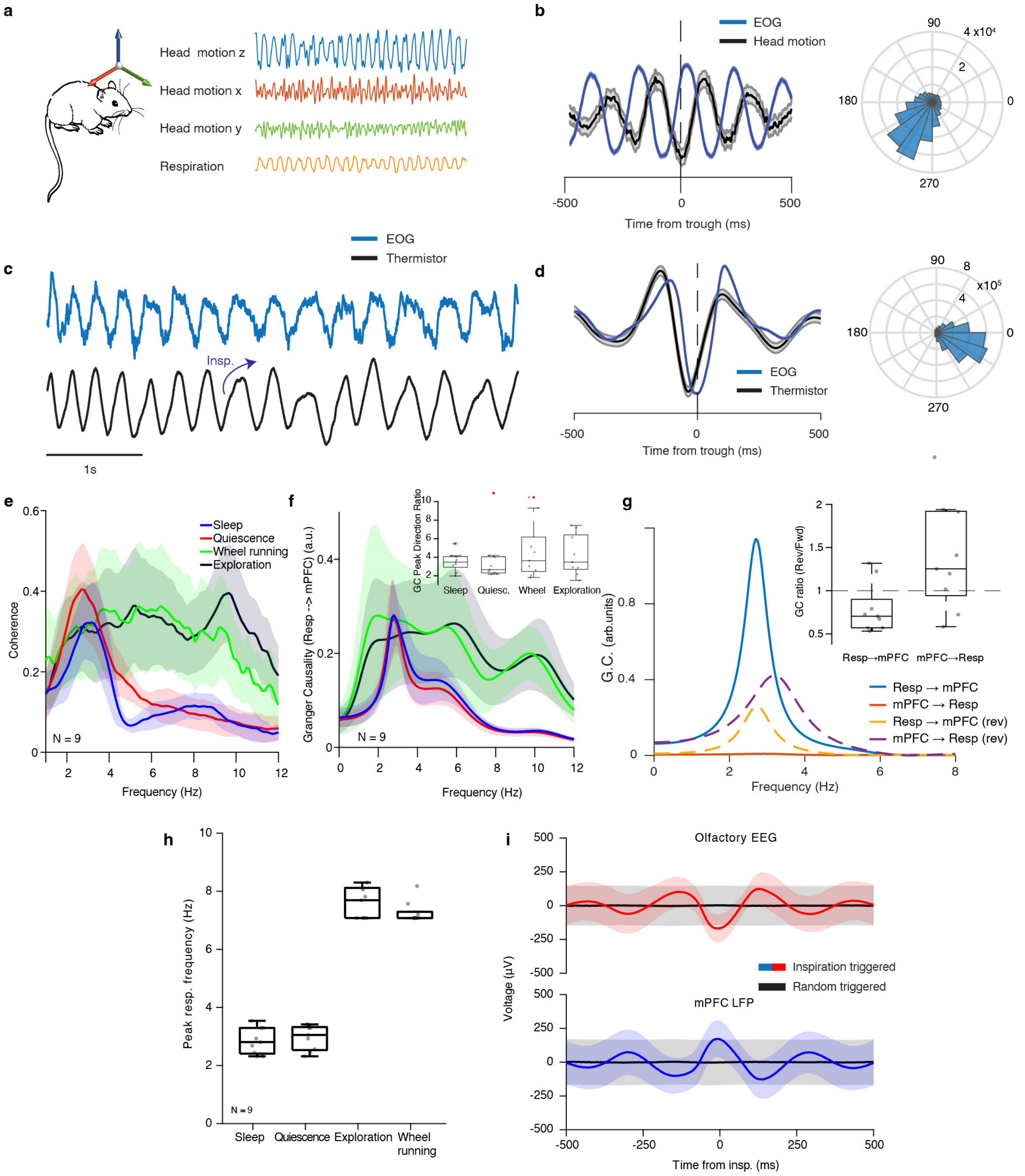

Supplementary Fig. 1 Validation of olfactory EEG recordings.
(**a**) Schematic and example head-motion and respiration traces from freely-behaving mice. (**b**) Left, head-motion trough-triggered olfactory EEG and head-motion signals during quiescence and sleep. Note the reliable phase relationship between the two signals. Right, distribution of phase shift between olfactory EEG and head-motion signals. (**c**) Example traces of olfactory EEG and simultaneous thermistor measurement. Thermistors are temperature-dependent resistors. When placed in or near the nasal cavity, they report the change in the temperature of the incident air flow due to breathing, since warmer air is exhaled and colder air inhaled[87]. (**d**) Olfactory EEG trough-triggered thermistor traces. Right, distribution of phase shift between the two signals. (**e**) Spectral estimation of coherence between prefrontal LFP and respiration for different behavioral states. (**f**) Spectrally resolved Granger causality from respiration to mPFC LFP for different behavioral states. Inset, quantification of the Granger causality measure ratio between the two directions (Forward/Reverse) for the peak frequency for each behavioral state (N = 9 mice). (**g**) Time-reversal control for the effect of signal-to-noise (SNR) differences on Granger causality estimates. If the effect of GC is mediated by the higher SNR of one signal compared to the other, reversing the signals in time should fully reverse the directionality of the calculated causality. In contrast, we observe that the calculated GC is higher for the forward (original) direction, compared

to the reversed signals, suggesting that this is due to the underlying Granger causal relationship between the two signals. Inset, quantification of the reverse to forward time calculation ratio for the two Granger causality directions (N = 9 mice). (**h**) Average respiration frequency in different behavioral states for all subjects (N = 9 freely-behaving mice). (**i**) Mean olfactory EEG and mPFC LFP triggered on the inspiration onset, compared to a randomized control. Shaded areas, mean ± SD. For box plots, the middle, bottom, and top lines correspond to the median, bottom, and top quartile, and whiskers to lower and upper extremes minus bottom quartile and top quartile, respectively. Shaded areas, mean ± SEM, unless otherwise noted. Source data are provided as a Source Data file.

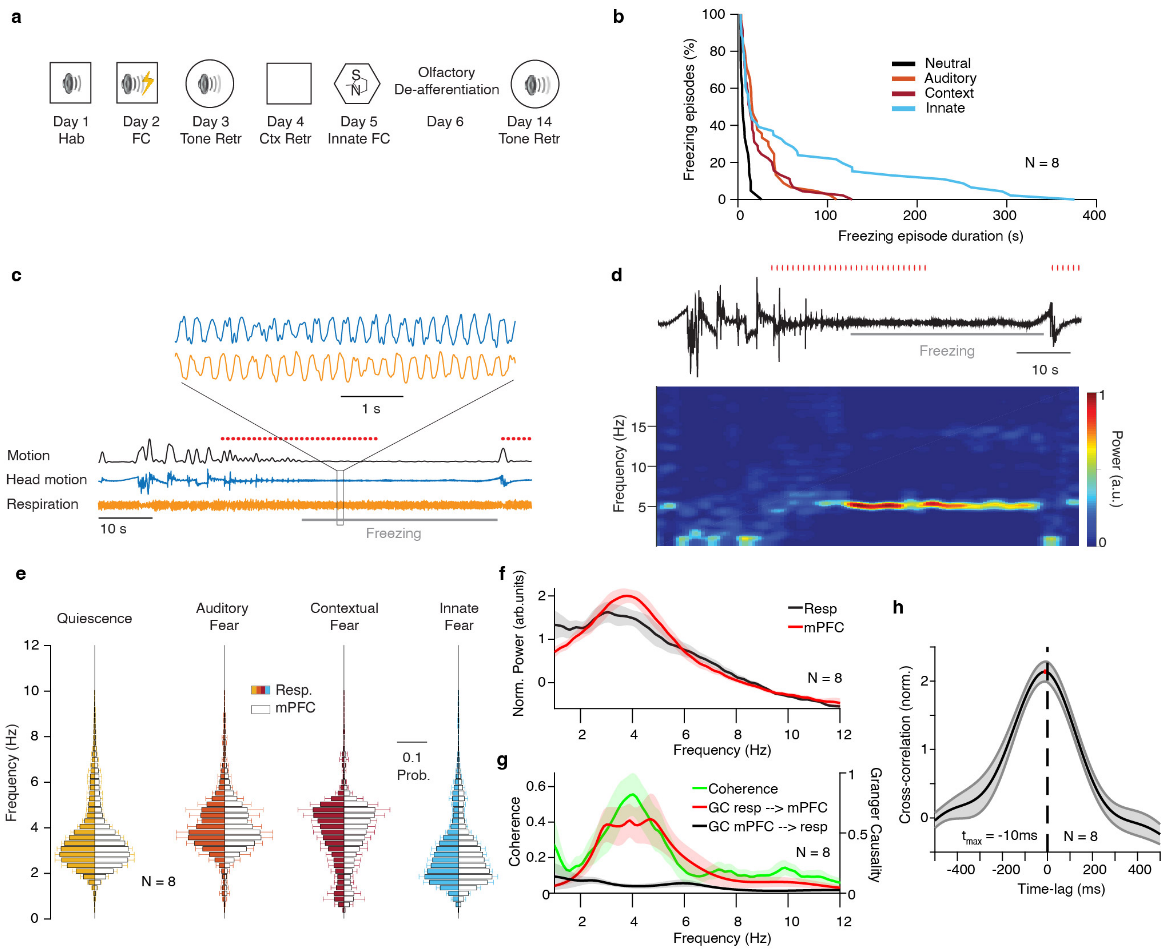

Supplementary Fig. 2 Respiratory dynamics during fear behavior.
(**a**) Experimental protocol. (**b**) Cumulative distribution of freezing episode duration during neutral context exploration (black trace) and different types of fear behavior for all animals (N = 8 freely-behaving mice). (**c**) Example compound motion, head-motion, and respiration traces during tone retrieval (day 3). Red ticks indicate CS presentations and the gray line indicates freezing episode duration. Inset, magnified traces during freezing reveals the presence of ∼4 Hz respiration-related oscillatory patterns in olfactory EEG and head-motion. (**d**) Example head-motion trace (top) and spectral decomposition of this signal (bottom) during freezing behavior, revealing the presence of strong oscillatory components during freezing behavior. Red ticks indicate CS presentations and the gray line indicates freezing episode duration. (**e**) Distribution of peak frequency bins of the spectrally decomposed respiration (left; darker colors) and mPFC LFP (right; lighter colors) during quiescence, auditory fear retrieval, contextual fear retrieval, and innate fear (N = 8 freely-behaving mice). (**f, g**) Spectral power (f), coherence (G - green trace), and Granger causality (g - red and black traces) for prefrontal LFP and respiration during freezing episodes (N = 8 mice). (**h**) 4Hz amplitude time-lagged cross-correlation between prefrontal LFP and respiration is consistent with the directionality indicated by Granger causality ($t_{max} = -10ms$) (N = 8 mice). Hab., habituation; FC, fear conditioning; Ret., retrieval; Ctx Retr., Context retrieval. Shaded areas, mean ± SEM.

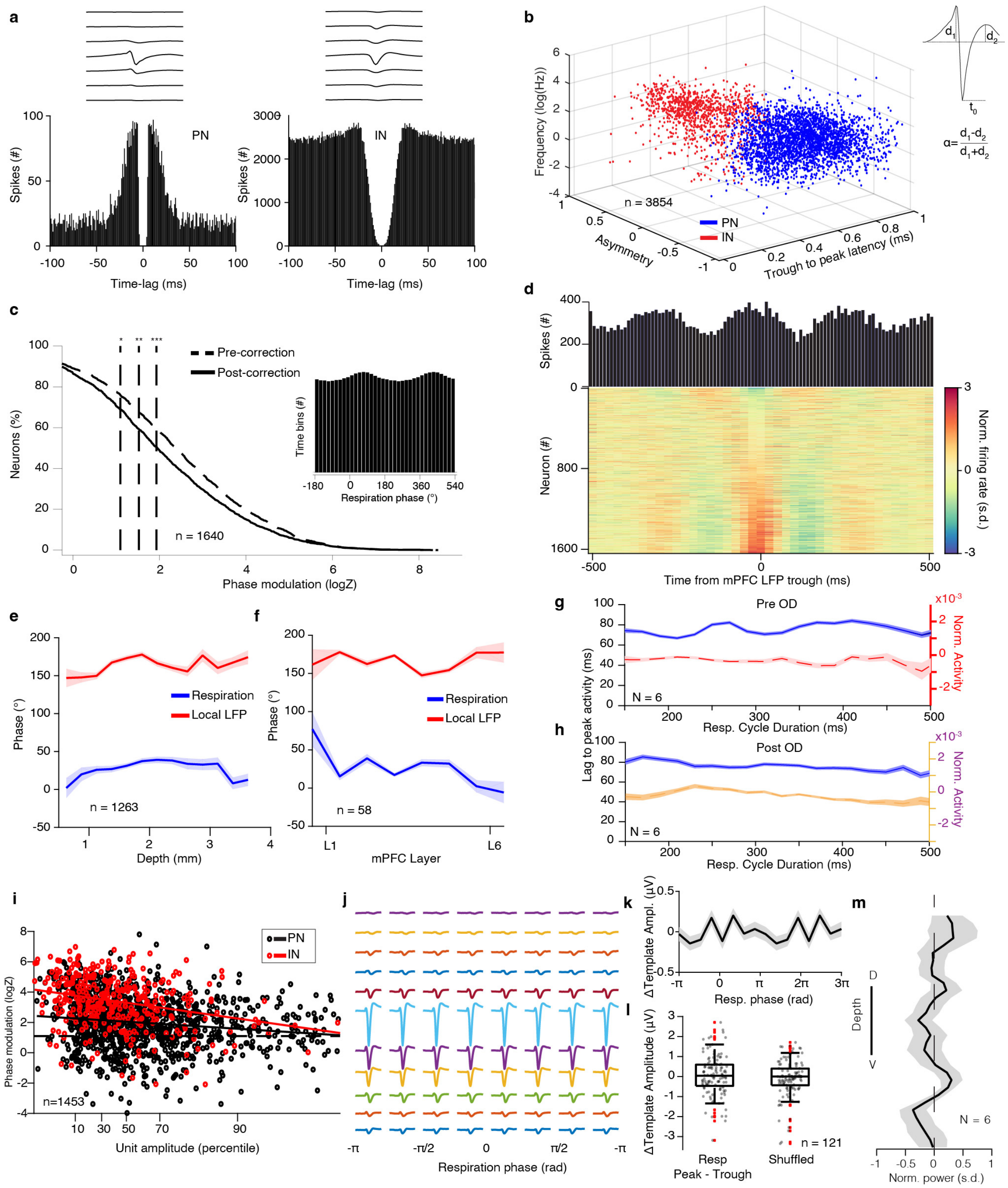

Supplementary Fig. 3 Unit classification and respiratory modulation.
(**a**) Average spike spatio-temporal waveforms (top) and auto-correlation histograms (bottom) for an example prefrontal PN (left) and IN (right). (**b**) Neurons from all structures were classified as putative principal cells and interneurons using an unsupervised clustering method based on the firing frequency, the trough to peak latency (t_0) and the waveform asymmetry index (α). (**c**) Cumulative distribution of the modulation strength (logZ) for all prefrontal neurons (n = 1640 cells), before and after correction for the non-uniformity of the respiratory phase. Uncorrected values are artifactually higher. Inset, example non-uniform prior distribution of the respiratory phase. (**d**) Top, example inspiration-triggered time histogram for an example prefrontal neuron. Bottom, color-coded normalized inspiration-triggered time histograms for all prefrontal neurons (n = 1640 cells), ordered by increasing normalized firing rate. (**e**) Depth-resolved average preferred phase for all prefrontal neurons (n = 1263 cells; N = head-fixed 11 mice), assessed in relation to the phase of the respiration and the phase of the local LFP. (**f**) Translaminar average preferred phase for all prefrontal neurons (n = 58 cells; N = 6 head-fixed mice), assessed in relation to the phase of the respiration and the phase of the local LFP. (**g**) Time-lag and peak normalized activity of the mPFC neurons after inspiration for inspiration events

giving rise to respiration cycles with different duration reveals no systematic relationship of these variables. (**h**) Same as (g) but after pharmacological OD. (**i**) Phase modulation magnitude (logZ) for each mPFC neuron is plotted against the relative amplitude for the unit for PN and IN. (**j**) Spike-waveform template across channels calculated for spikes occurring at different respiration phases for an example mPFC neuron. (**k**) Distribution of maximal amplitude differences across channels for spike-waveform templates occurring at different respiration phases. (**l**) Distribution of maximal template amplitude differences for templates from the peak and trough of the respiration compared with differences from a random subset of template phases (as in (k)) ($n = 1453$ cells). (**m**) Average depth-resolved z-score normalized mPFC LFP power in the 2-5 Hz band following OD ($N = 6$ head-fixed mice). Refer to Fig. 2g for the same analysis before OD. Shaded areas, mean $\pm$ SEM. Stars indicate significance levels (* $P<0.05$; ** $P<0.01$; *** $P<0.001$). s.d., standard deviations. M2, motor area 2; ACC, anterior cingulate cortex; PL, prelimbic; IL, infralimbic; DP, dorsal peduncular; L1, layer 1; L6, layer 6. For box plots, the middle, bottom, and top lines correspond to the median, bottom, and top quartile, and whiskers to lower and upper extremes minus bottom quartile and top quartile, respectively.

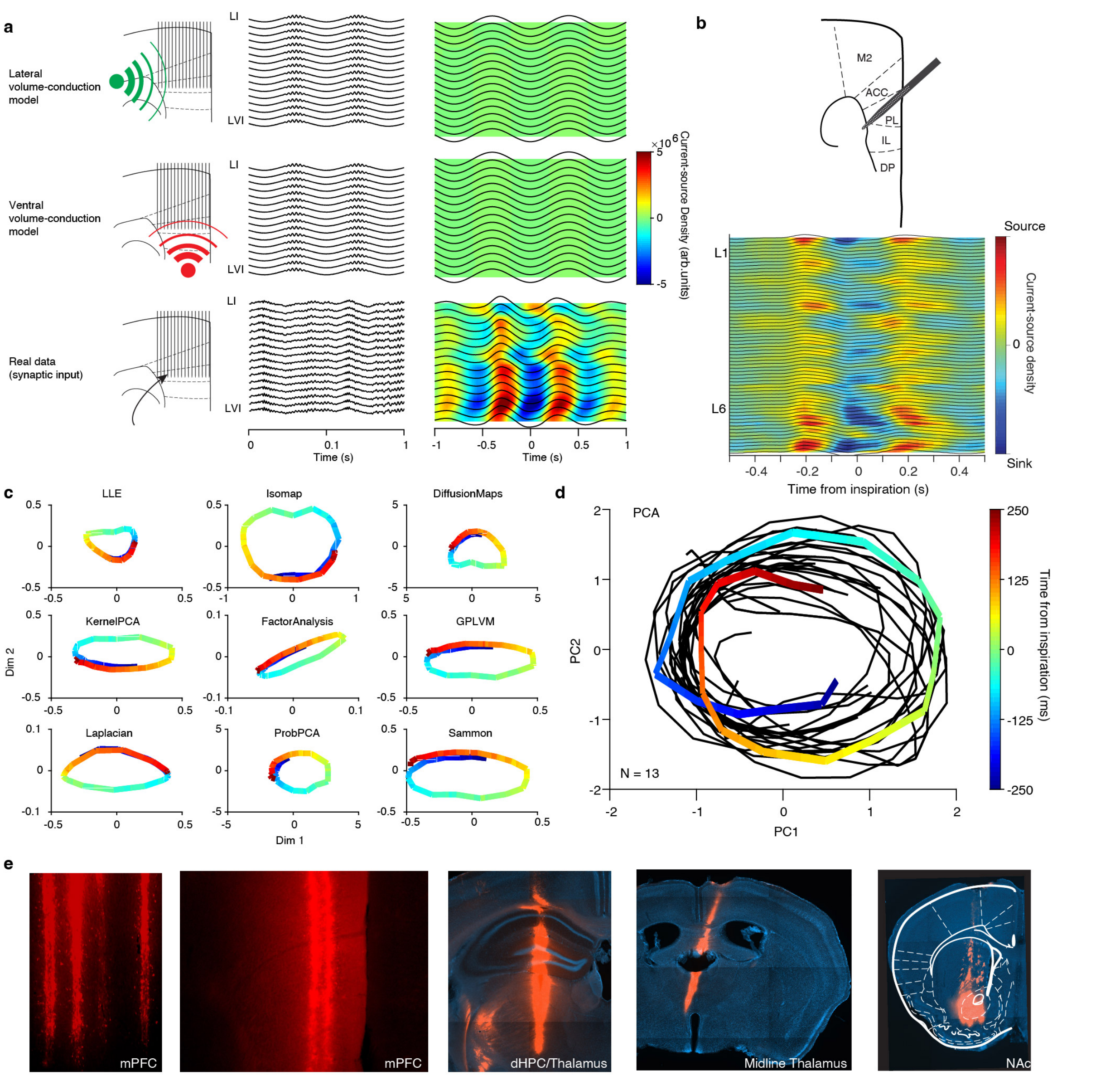

Supplementary Fig. 4 Volume conduction, CSD, and dimensionality-reduction controls.
(**a**) Comparison of LFP signals and CSD calculation for two possible volume conduction models (lateral and ventral oscillatory source). (**b**) Top, schematic of a single-shank angled high-density polytrode inserted in the dorsal mPFC. Bottom, Example high--resolution inspiration-triggered CSD of the mPFC respiration-related oscillation using a single-shank angled polytrode used to confirm the observations based on data acquired with the multi-shank silicon probe shown in Fig. 2l,m. (**c**) Comparison of multiple dimensionality-reduction methods on the same example data, to confirm the generality and reliability of this approach for identifying manifolds of inspiration-triggered neuronal activity. (**d**) Trajectory of the inspiration-triggered neuronal population activity in the PC space for each animal (black lines) and across all animals (color-coded line). (**e**) Example histological verification of recording configurations in the mPFC, dCA1 and somatic thalamus, midline thalamus, and NAc.

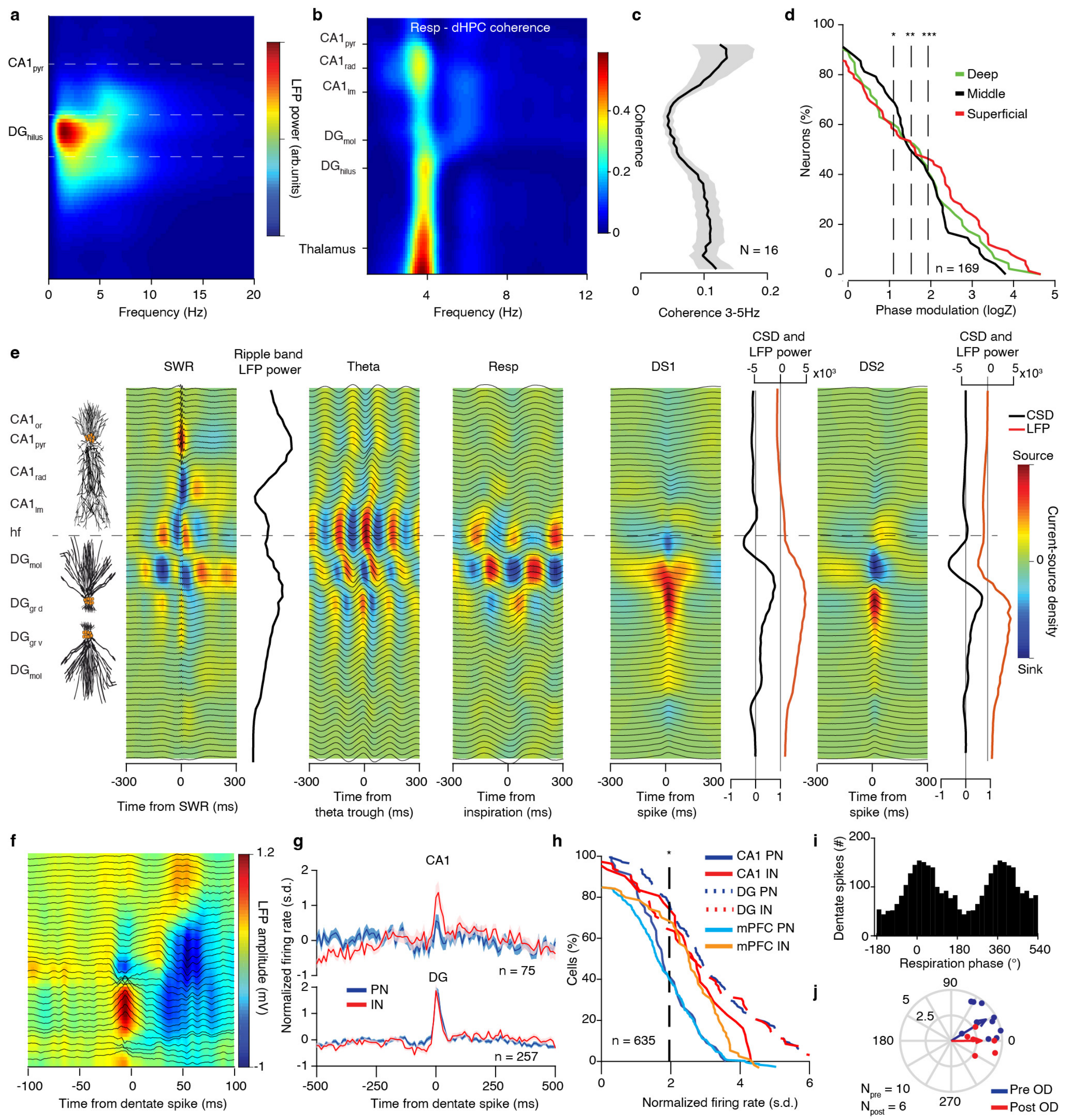

Supplementary Fig. 5 Translaminar profile of hippocampal dynamics.
(**a**) Example translaminar profile of spectral power of the hippocampal LFP during quiescence and slow-wave sleep. Note the strong peak in 2-4 Hz power reflecting DG LFP entrained by respiration. (**b**) Example translaminar profile of coherence of the hippocampal LFP with respiration. (**c**) Average coherence across hippocampal layers for the population (N = 16 mice). (**d**) Cumulative distribution of the respiratory phase modulation strength for all CA1 putative pyramidal cells (n = 169 neurons), grouped based on their location within the pyramidal layer into deep (n = 46 cells), middle (n = 48 cells) and superficial (n = 75 cells) cells. (**e**) Left, schematic depiction of the neuronal alignment of CA1 pyramidal cells and DG granule cells, aligned to the CSD profiles as described in the Methods section. Right, example CSD profiles of the dorsal hippocampus of the same animal, triggered on the peak of sharp-wave ripple (SWR) events, the troughs of theta oscillations, inspiratory events and the two types of dentate spikes (DS1 and DS2). The power of the ripple-band and the CSD magnitude and LFP power for the two types of dentate spikes is aligned to the CSD profiles. Horizontal dashed line indicates the hippocampal fissure. Since there are no aligned dendrites in the hilus proper to give rise to active sinks, we interpret the sinks in the molecular layer as active, arising from MEC and LEC inputs, while the source in the hilar proper is a passive return current of sources in granular layer[39]. The proximity to the hilus apex and the smoothing result in a merge of two ventral and dorsal dipoles together, but active sinks below ventral and matching above dorsal blades can be clearly identified. The sink alternation with this hilar source in the hilar proper is likely, not real but relates to the AC-coupled nature of recordings which produces an alternating source/sink for each true DC CSD sink/source[12,83]. (**f**) Color-coded LFP power depth profile and overlaid LFP traces of an example dentate spike. (**g**) Average

dentate spike-triggered normalized spiking activity for all CA1 (n = 75 cells) (top) and DG (bottom) PNs and INs (n = 257 cells). (**h**) Cumulative distribution of the dentate spike-triggered normalized firing of CA1, DG and mPFC PNs (CA1, n = 44 cells; DG, n = 175 cells; mPFC, n = 239 cells) and INs (CA1, n = 31 cells; DG, n = 82 cells; mPFC, n = 64 cells). (**i**) Respiration phase histogram for dentate spikes in one example animal. (**j**) Preferred respiratory phase of dentate spike occurrence and phase modulation strength for all animals (dots) and population average (arrows) before and after OD (n_{pre} = 10 mice,n_{post} = 6 mice). Stars indicate significance levels (* P<0.05; ** P<0.01; *** P<0.001). $CA1_{or}$, oriens; $CA1_{pyr}$, str. pyramidale; $CA1_{rad}$, str. radiatum; $CA1_{lm}$, str. lacunosum-moleculare; hf, hippocampal fissure; DG_{mol}, dentate gyrus – molecular layer; DG_{om}, outer molecular layer; DG_{mm}, middle molecular layer; $DG_{grd/v}$, granule cell layer (dorsal/ventral); DG_{hil}, hilus. Shaded areas, mean ± SEM.

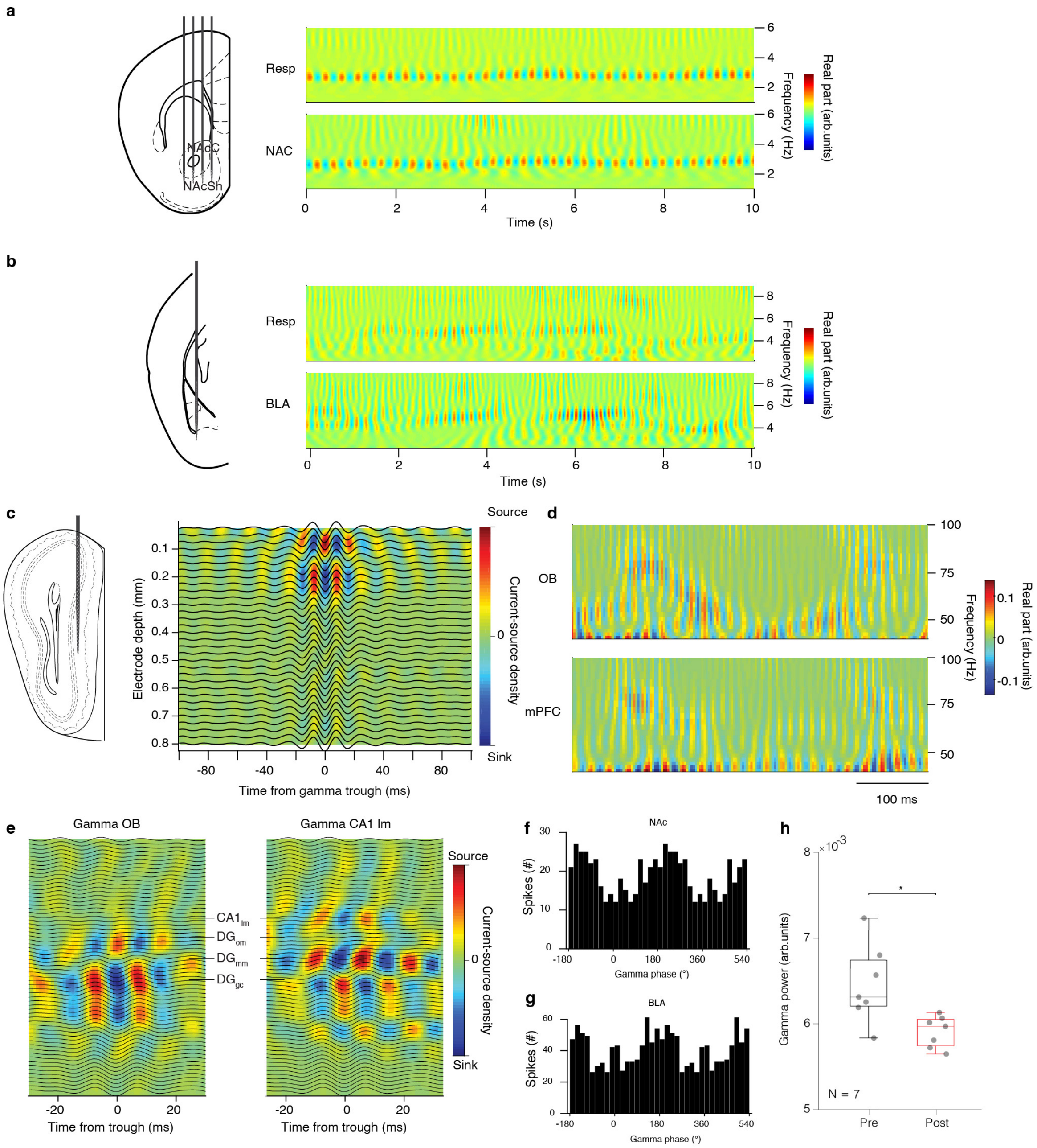

Supplementary Fig. 6 Gamma dynamics.
(**a**) Left, schematic of a recording from the NAc using a 4 shank silicon polytrode. Right, example spectral representation of the respiration and NAc LFP using the real part of the wavelet transform highlights the reliable phase relationship between the two signals. (**b**) Left, schematic of a high-density polytrode recording from the BLA. Right, example spectral representation of the respiration and BLA LFP using the real part of the wavelet transform highlights the reliable phase relationship between the two signals. (**c**) Left, schematic of a high-density polytrode recording across layers of the olfactory bulb. Right, example high-resolution inspiration-triggered CSD of the olfactory bulb. (**d**) Example spectral representation of the OB and mPFC gamma-band LFP using the real part of the wavelet transform highlights the transient phase relation between bursts of gamma oscillations in the two structures. (**e**) Example olfactory bulb gamma- and CA1lm gamma-triggered LFP traces and translaminar CSD in the dorsal hippocampus. (**f**) Gamma phase distribution of the spikes of an example NAc neuron. (**g**) Gamma phase distribution of the spikes of an example BLA neuron. (**h**) Gamma power in the mPFC before and after OD. (N = 7 mice, Wilcoxon signed-rank two-sided test, pre- versus post-OD, P = 0.0312). arb. units, arbitrary units. Star indicates significance level (* P<0.05). OD, olfactory deafferentation. For box plots, the middle, bottom, and top lines correspond to the median, bottom, and top quartile, and whiskers to lower and upper extremes minus bottom quartile and top quartile, respectively. Source data are provided as a Source Data file.

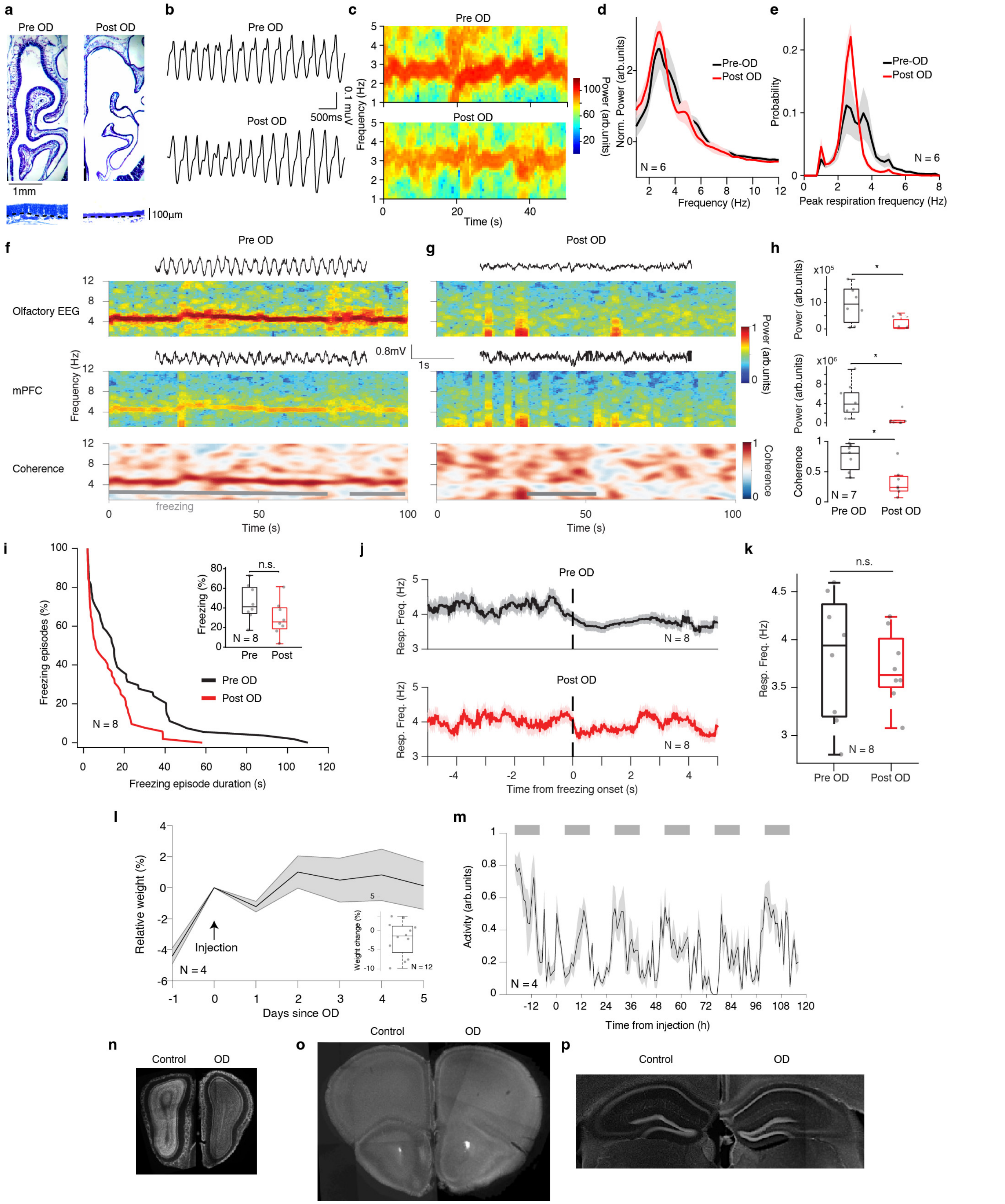

Supplementary Fig. 7 Effect of olfactory deafferentation on fear behavior.
(**a**) Example histological verification of the effectiveness of the experimental strategy for the olfactory deafferentation. (**b**) Example thermistor traces of the respiration signal before and after OD. (**c**) Example spectral decomposition of the respiration signal before and after OD. (**d**) Average spectral power of the respiration signal before and after OD (N = 6 head-fixed mice). (**e**) Distribution of the peak respiration frequency before and after OD (N = 6 head-fixed mice). (**f, g**) Example spectral decomposition of the respiration (top) and mPFC LFP (middle) during freezing behavior and coherogram between the two signals (bottom), in baseline conditions (a) and after OD (b). (**h**) Average olfactory EEG (top) and mPFC LFP (middle) power and coherence between them (bottom) for the 2-5 Hz band before and after OD (N = 7 freely-behaving mice, Wilcoxon signed-rank two-sided tests in all plots,

pre- versus post-OD, P = 0.0156 (top), P = 0.0156 (middle), P = 0.0469 (bottom)). (**i**) Cumulative distribution of freezing episode duration for tone retrieval before and after OD (N = 8 freely-behaving mice). Inset, average freezing before and after OD (N = 8 mice, Wilcoxon signed-rank two-sided test, pre- versus post-OD, P = 0.109). (**j**) Freezing onset-triggered respiratory frequency before and after OD (N = 8 mice). (**k**) Average respiratory frequency during freezing, before and after OD (N = 8 mice, Wilcoxon signed-rank two-sided test, pre- versus post-OD, P = 0.742). (**l**) Relative weight change of mice following methimazole injection (N = 4 mice). Inset, weight change on day 3 post OD (N = 12 mice, Wilcoxon signed-rank two-sided test, pre- versus post-OD, P = 0.198). (**m**) Activity profile of mice following OD exhibits normal circadian patterns (N = 4 freely-behaving mice). Gray bars on top indicate the dark phase of the lighting cycle. (**n-p**) Example histological reconstruction of the OB, mPFC, hippocampus 1 week after methimazole injection or saline control. For box plots, the middle, bottom, and top lines correspond to the median, bottom, and top quartile, and whiskers to lower and upper extremes minus bottom quartile and top quartile, respectively. Stars indicate significance levels (* $P<0.05$; ** $P<0.01$; *** $P<0.001$). n.s., not significant; OD, olfactory deafferentation. Shaded areas, mean ± SEM. Source data are provided as a Source Data file.

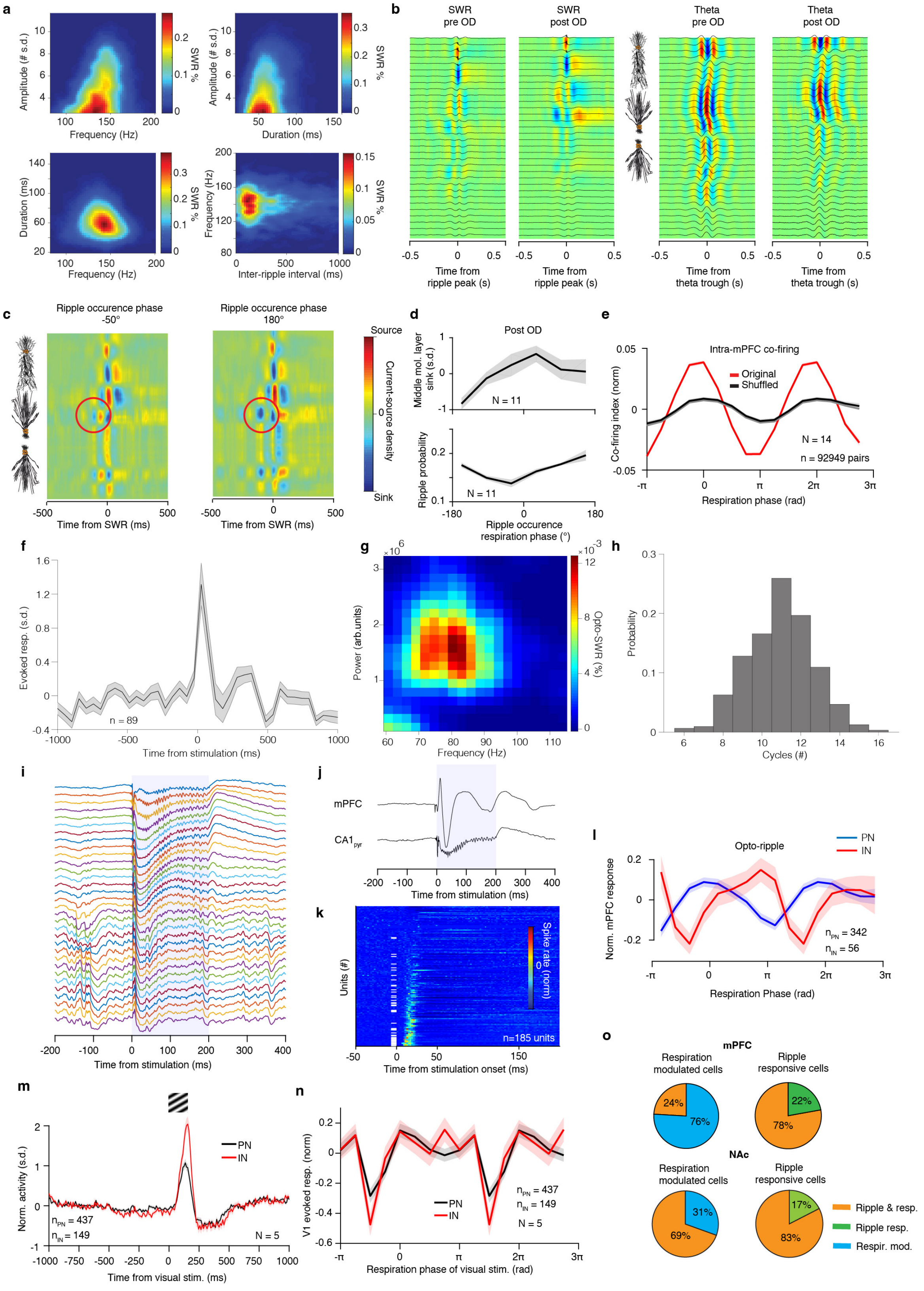

Supplementary Fig. 8 Effect of OD on characteristics of hippocampal respiration-related patterns. (**a**) Example color-coded pairwise joint probability distributions of the properties of ripple events, including the amplitude, frequency, duration, and inter-ripple interval. (**b**) Example current-source density profiles of the dorsal hippocampus of the same animal,

triggered on the peak of sharp-wave ripple (SWR) events and the troughs of theta oscillations (detected in CA1 pyramidal layer) before and after OD. (**c**) Example ripple-triggered translaminar current-source density profiles of the dorsal hippocampus grouped by the respiratory phase of the ripple occurrence: -50°(left) and 180°(right). Note strong sink at DG middle mol. Layer (red circle) present in the latter but not former. (**d**) Average respiration phase-resolved ripple-triggered middle molecular layer dentate sink magnitude (left) and corresponding phase ripple normalized incidence rate (right) (N = 11 mice). (**e**) Average normalized co-firing index for all pairs of mPFC cells (n = 92949, N = 14 freely-behaving and head-fixed mice) compared to shuffle control. (**f**) Evoked response (z-score normalized) of dCA1 neurons in response to the optogenetic stimulation used for opto-ripple generation (n=89 cells). (**g**) Joint distribution of frequency and power of optogenetically induced ripples. (**h**) Probability distribution of number of cycles of optogenetically induced ripples. (**i**) Translaminar profile of dCA1 LFP during square-pulse optogenetic stimulation reveals a sharp excitation/inhibition at the onset and the gradual relaxation of the system into a fast oscillatory mode. (**j**) Example mPFC and dCA1 pyramidal layer LFP for a square-pulse stimulation. (**k**) Prefrontal unit response to square-pulse stimulation of the dCA1. White bars indicate putative inhibitory neurons. (**l**) Prefrontal evoked activity for PN (blue traces) and IN (red traces) in response to optogenetically-generated ripples, as a function of the respiratory phase of occurrence of the opto-ripple (n = 398 cells). (**m**) Average response of V1 PN and IN cells to the presentation of visual stimulus (n = 586 cells, N = 5 mice). (**n**) Phase modulation of the V1 evoked response by the phase of the respiration during the stimulus presentation (n = 586 cells, N = 5 mice). (**o**) Pie charts indicating the percentage of mPFC neurons being either phase modulated by respiration (resp. mod), responding significantly to ripples (ripple resp.) or being both significantly modulated by the respiratory rhythm and significantly responsive to ripples for all mPFC and NAc respiration-modulated cells and ripple-responsive cells. Shaded areas, mean ± SEM. Stars indicate significance levels (* $P<0.05$; ** $P<0.01$; *** $P<0.001$). OD, olfactory deafferentation.

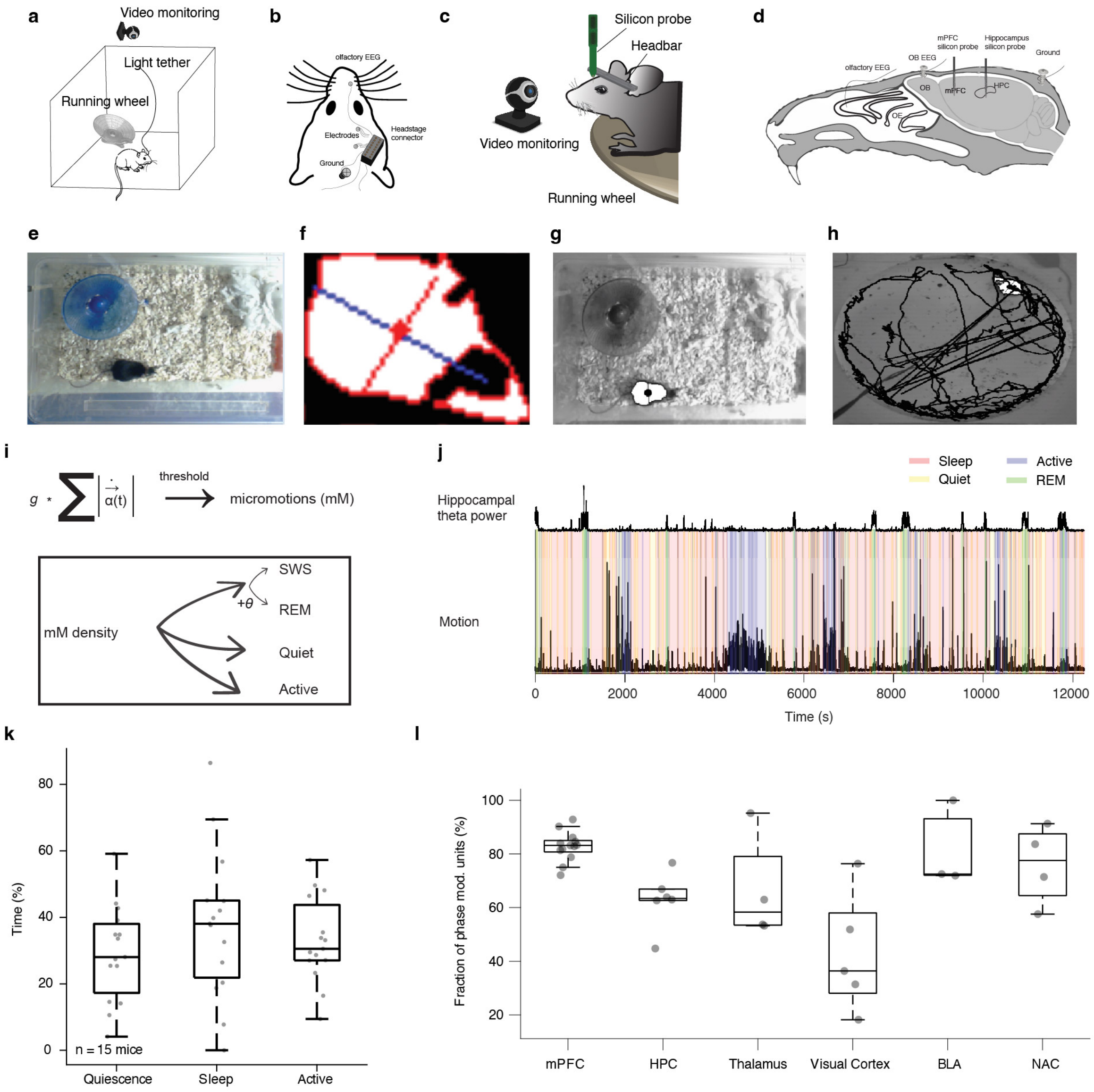

Supplementary Fig. 9 configuration and behavioral segmentation
(**a**) Schematic description of the recording configuration in the homecage of freely-behaving mice. (**b**) Schematic description of the electrode implantation and configuration for the recordings from freely-behaving mice. (**c**) Schematic description of the recording configuration for head-fixed mice. (**d**) Schematic description of a typical silicon probe configuration for the recordings from head-fixed mice. (**e**) Overhead camera view of a typical recording session in the homecage. (**f**) Mouse body shape extraction and identification of the center of mass and major and minor axes. (**g**) Overlay of the detected mouse body on the overhead camera image. (**h**) Cumulative trace of the mouse location in an open arena exploration session. (**i**) Algorithm for the detection and classification of different behavioral states using behavioral variables extracted from the head-motion in freely-behaving mice or face motion in head-fixed mice and based on the calculation of micromotion (mM) density and hippocampal theta power. (**j**) Example classification of behavioral states from a recording in the home cage, using compound motion and hippocampal theta power. (**k**) Relative time spent in quiescence, sleep, or active periods for each subject (N = 15 head-fixed mice). (**l**) Fraction of neurons significantly phase modulated by the breathing for each brain region (N = 13 mice for mPFC, N = 6 mice for HPC, N = 4 mice for Thalamus, N = 5 for Visual Cortex, N = 3 for BLA, N = 4 for NAc.). For box plots, the middle, bottom, and top lines correspond to the median, bottom, and top quartile, and whiskers to lower and upper extremes minus bottom quartile and top quartile, respectively. Source data are provided as a Source Data file.
