# Supplementary material for: Breathing coordinates cortico-hippocampal dynamics in mice during offline states
Source: Nat Commun. 2022 Jan 24;13:467. doi: 10.1038/s41467-022-28090-5 (PMC8786964; doi:10.1038/s41467-022-28090-5)
Supplement: Supplementary file 3 — Reporting Summary [file 41467_2022_28090_MOESM3_ESM.pdf]

nature portfolio

Corresponding author(s): Anton Sirota

Last updated by author(s): Nov 30, 2021

Reporting Summary

Nature Portfolio wishes to improve the reproducibility of the work that we publish. This form provides structure for consistency and transparency in reporting. For further information on Nature Portfolio policies, see our Editorial Policies and the Editorial Policy Checklist.

Statistics

For all statistical analyses, confirm that the following items are present in the figure legend, table legend, main text, or Methods section.

n/a	Confirmed	
☐	☒	The exact sample size (*n*) for each experimental group/condition, given as a discrete number and unit of measurement
☒	☐	A statement on whether measurements were taken from distinct samples or whether the same sample was measured repeatedly
☐	☒	The statistical test(s) used AND whether they are one- or two-sided<br>*Only common tests should be described solely by name; describe more complex techniques in the Methods section.*
☐	☒	A description of all covariates tested
☐	☒	A description of any assumptions or corrections, such as tests of normality and adjustment for multiple comparisons
☐	☒	A full description of the statistical parameters including central tendency (e.g. means) or other basic estimates (e.g. regression coefficient) AND variation (e.g. standard deviation) or associated estimates of uncertainty (e.g. confidence intervals)
☐	☒	For null hypothesis testing, the test statistic (e.g. *F*, *t*, *r*) with confidence intervals, effect sizes, degrees of freedom and *P* value noted<br>*Give P values as exact values whenever suitable.*
☒	☐	For Bayesian analysis, information on the choice of priors and Markov chain Monte Carlo settings
☒	☐	For hierarchical and complex designs, identification of the appropriate level for tests and full reporting of outcomes
☐	☒	Estimates of effect sizes (e.g. Cohen's *d*, Pearson's *r*), indicating how they were calculated

Our web collection on statistics for biologists contains articles on many of the points above.

Software and code

Policy information about availability of computer code

Data collection	OpenEphys GUI (v0.4 - v0.5), Bonsai (v2.0 - v2.6)
Data analysis	Matlab 2014 - 2019b, open source Neuroscope, Ndmanager processing utilities; open source Mountainsort and Kilosort2 spike sorting software, open source Chronux, MVGC toolboxes, custom code (https://github.com/nikolaskaralis/Karalis2021_NatureComm)

For manuscripts utilizing custom algorithms or software that are central to the research but not yet described in published literature, software must be made available to editors and reviewers. We strongly encourage code deposition in a community repository (e.g. GitHub). See the Nature Portfolio guidelines for submitting code & software for further information.

Data

Policy information about availability of data

All manuscripts must include a data availability statement. This statement should provide the following information, where applicable:

- Accession codes, unique identifiers, or web links for publicly available datasets
- A description of any restrictions on data availability
- For clinical datasets or third party data, please ensure that the statement adheres to our policy

Relevant data that support the findings of this study are available from the authors upon reasonable request. Source data are provided with this paper.

Field-specific reporting

Please select the one below that is the best fit for your research. If you are not sure, read the appropriate sections before making your selection.

☒ Life sciences ☐ Behavioural & social sciences ☐ Ecological, evolutionary & environmental sciences

For a reference copy of the document with all sections, see nature.com/documents/nr-reporting-summary-flat.pdf

Life sciences study design

All studies must disclose on these points even when the disclosure is negative.

Sample size	Sample sizes for animals and neurons used for phase modulation analyses were chosen based on established procedures in the field and were selected to be sufficient to determine significance both in behavior tests and electrophysiological recordings. The numbers were calculated based on the difficulty of the recordings performed from each brain region and the recording yield (number of units recorded per session). For the olfactory deafferentation experiments, the same mice were used (paired analysis - before vs after), allowing lower sample size.
Data exclusions	All animals with sufficient quality of electrophysiology recordings were included in the study. No data that were applicable for each analysis were excluded.
Replication	There was no replication carried out in this study due to the large numbers of animals and experimental conditions/measurements required to complete the analysis. However, we ensured results are consistent across batches of mice, serving as internal replication. Some effects reported in the manuscript (entrainment of ripples by respiration, LFP coherence to respiration) have been assessed both in chronic mice and head-fixed mice.
Randomization	For each animal batch, animals were randomly allocated into experimental groups. We followed this approach in order to avoid unintentional bias from the evolving surgical skills or batch of animals.
Blinding	All data analysis was performed in automatic regime across all sessions regardless of conditions, i.e. processing and quantification throughout the analysis was blind. For the data collection, blinding was not possible for the olfactory deafferentation experiments (before vs after condition) and was not relevant for the conclusions drawn.

Reporting for specific materials, systems and methods

We require information from authors about some types of materials, experimental systems and methods used in many studies. Here, indicate whether each material, system or method listed is relevant to your study. If you are not sure if a list item applies to your research, read the appropriate section before selecting a response.

Materials & experimental systems

n/a	Involved in the study	
☒	☐	Antibodies
☒	☐	Eukaryotic cell lines
☒	☐	Palaeontology and archaeology
☐	☒	Animals and other organisms
☒	☐	Human research participants
☒	☐	Clinical data
☒	☐	Dual use research of concern

Methods

n/a	Involved in the study	
☒	☐	ChIP-seq
☒	☐	Flow cytometry
☒	☐	MRI-based neuroimaging

Animals and other organisms

Policy information about studies involving animals; ARRIVE guidelines recommended for reporting animal research

Laboratory animals	Mus musculus, C57BL/6J, male, 8-20 weeks old.<br>Naive male C57BL6/J mice (3 months old, Jackson Laboratory) were individually housed for at least a week before all experiments, under a 12 light-dark cycle, ambient temperature 22C, 50% humidity, and provided with food and water ad libitum.
Wild animals	No wild animals were used
Field-collected samples	This study did not require samples collected from the field
Ethics oversight	Electrophysiological experiments with C57BL/6J mice were carried out under the German Law for Protection of Animals (TierSchG) and were approved by the local authorities (Regierung von Oberbayern - ROB-55.2-2532.Vet_02-16-170).

Note that full information on the approval of the study protocol must also be provided in the manuscript.
